# Social determinants of health and disparities in pediatric trauma care: protocol for a systematic review and meta-analysis

**DOI:** 10.1186/s13643-024-02510-7

**Published:** 2024-03-22

**Authors:** Janyce Eunice Gnanvi, Natalie Yanchar, Gabrielle Freire, Emilie Beaulieu, Pier-Alexandre Tardif, Mélanie Bérubé, Alison Macpherson, Ian Pike, Roger Zemek, Isabelle J. Gagnon, Sasha Carsen, Belinda Gabbe, Soualio Gnanou, Cécile Duval, Lynne Moore

**Affiliations:** 1 Population Health and Optimal Health Practices Unit, Trauma-Emergency-Critical Care Medicine, Centre de Recherche du CHU de Québec-Université Laval (Hôpital de l’Enfant-Jésus), Université Laval, Montreal, QC Canada; 2 Department of Social and Preventive Medicine, Faculté de Médecine, Université Laval, Quebec City, QC Canada; 3 Department of Surgery, University of Calgary, Calgary, AB Canada; 4 Division of Emergency Medicine, Department of Pediatrics, Faculty of Medicine, University of Toronto, Toronto, ON Canada; 5 Department of Pediatrics, Faculté de Médecine, Centre Hospitalier Universitaire de Québec, Université Laval, Quebec City, QC Canada; 6 Faculty of Nursing, Université Laval, Quebec City, QC Canada; 7 Faculty of Health, York University, Toronto, ON Canada; 8 Department of Pediatrics, BC Injury Research and Prevention Unit, The University of British Columbia, Vancouver, BC Canada; 9 Department of Pediatrics, Children’s Hospital of Eastern Ontario, Ottawa, ON Canada; 10 Division of Pediatric Emergency Medicine, McGill University Health Centre, Montreal Children’s Hospital, Montreal, QC Canada; 11 Division of Orthopaedic Surgery, Children’s Hospital of Eastern Ontario, Ottawa, ON Canada; 12 School of Public Health and Preventive Medicine, Monash University, Melbourne, VIC Australia

**Keywords:** Disparities, Inequities, Social determinants of health, PROGRESS-plus, Pediatrics, Injury care, Trauma care, Systematic review, Meta-analysis

## Abstract

**Background:**

Social determinants of health (SDH), including “the conditions in which individuals are born, grow, work, live and age” affect child health and well-being. Several studies have synthesized evidence about the influence of SDH on childhood injury risks and outcomes. However, there is no systematic evidence about the impact of SDH on accessing care and quality of care once a child has suffered an injury. We aim to evaluate the extent to which access to care and quality of care after injury are affected by children and adolescents’ SDH.

**Methods:**

Using Cochrane methodology, we will conduct a systematic review including observational and experimental studies evaluating the association between social/material elements contributing to health disparities, using the PROGRESS-Plus framework: place of residence, race/ethnicity/culture/language, occupation, gender/sex, religion, education, socioeconomic status, and social capital and care received by children and adolescents (≤ 19 years of age) after injury. We will consult published literature using PubMed, EMBASE, CINAHL, PsycINFO, Web of Science, and Academic Search Premier and grey literature using Google Scholar from their inception to a maximum of 6 months prior to submission for publication. Two reviewers will independently perform study selection, data extraction, and risk of bias assessment for included studies. The risk of bias will be assessed using the ROBINS-E and ROB-2 tools respectively for observational and experimental study designs. We will analyze data to perform narrative syntheses, and if enough studies are identified, we will conduct a meta-analysis using random effects models.

**Discussion:**

This systematic review will provide a synthesis of evidence on the association between SDH and pediatric trauma care (access to care and quality of care) that clinicians and policymakers can use to better tailor care systems and promote equitable access and quality of care for all children. We will share our findings through clinical rounds, conferences, and publication in a peer-reviewed journal.

**Systematic review registration:**

PROSPERO CRD42023408467

**Supplementary Information:**

The online version contains supplementary material available at 10.1186/s13643-024-02510-7.

## Background

Social determinants of health (SDH) refer to the social, economic, and environmental factors that influence individuals’ health and well-being [1]. Inequities in SDH, including inequitable distributions of resources, opportunities, and power among different population groups, result in health disparities [1, 2], defined as “health differences that are closely linked with social or economic disadvantage” [3]. Research has shown that inequities in SDH not only shape disparities in health outcomes but also contribute to the exacerbation of these disparities through barriers to high-quality healthcare services such as limited access to resources and discriminatory practices [4, 5]. Populations facing these disparities experience heightened barriers to receiving timely, appropriate, and high-quality care, with increased health disparities and poorer health outcomes [5–7]. Addressing disparities in healthcare delivery is a critical step toward reducing health disparities and improving health outcomes for all individuals, particularly marginalized and underserved populations.

Injury is the leading cause of mortality and morbidity in children worldwide [8] with more than two-thirds of children reporting at least one injury by the age of 16 in the United States [9] and 31% of Canadian adolescents reporting an injury serious enough to limit their normal activities or require medical care in 2016 [10]. Extensive evidence, including systematic reviews with meta-analyses, supports the significant influence of SDH on the risk of childhood injury and subsequent health outcomes [11–13]. Studies have also investigated the impact of SDH-related inequities on healthcare delivery for injured children, recognizing that the accessibility and quality of care provided strongly shape health outcomes in this population [14–16]. These studies have consistently identified socioeconomic status (SES), race, ethnicity, insurance status, geographic location, and language barriers as key factors associated with disparities in the delivery of healthcare following pediatric injury [17, 18]. However, this evidence has not been systematically reviewed. Our objective was therefore to synthesize current evidence on the influence of SDH on the delivery of acute healthcare for children and adolescents following injury using the PROGRESS-Plus framework.

## Methods

This systematic review will be conducted according to Cochrane methodology [19], and the protocol is reported in line with the Preferred Reporting Items for Systematic Reviews and Meta-Analyses-Protocols (PRISMA-P) statement (Additional file 1) [20]. The protocol has been registered in the International Prospective Register of Systematic Reviews (PROSPERO CRD42023408467). This protocol was developed in collaboration with our project advisory committee including pediatric trauma physicians (emergency department (ED), intensive care, trauma surgery, orthopedic surgery), pediatric nurse practitioners, ED physicians in referral hospitals, and equity–diversity and inclusion experts.

## Eligibility criteria

We defined our eligibility criteria using the Population, exposure, comparator, outcomes, and study design (PECOS) approach [21].

### Populations

We will consider studies on children and adolescents (≤ 19 years of age) [22] who present to the ED or are admitted to the hospital following injury. We will include studies on the following injury mechanisms: motor vehicle collisions, falls, struck by/against, other transport, firearm, and cut/pierce [23–25]. As is common in injury research and because risk factors, presentation, clinical management, and prognosis are distinct, we will not include studies on injuries due to burns, foreign objects, poisoning, or late effects of injury. Therefore, studies in which more than 20% of the population is injured by these mechanisms will be excluded.

### Exposures

We have defined children’s SDH using the PROGRESS-Plus framework. The PROGRESS-Plus framework is a conceptual tool used in public health research and policy to systematically analyze and address health disparities. The framework is developed and endorsed by the Campbell and Cochrane Equity Methods Group [26]. Using this framework, we will include studies that assess healthcare delivery according to at least one of the following factors: children’s place of residence (e.g., geographical location, urbanicity); race/ethnicity/culture/language; occupation; gender/sex; religion; education; socioeconomic status (e.g., family income level, insurance status); and social capital. The “Plus” stands for other factors associated with discrimination, exclusion, marginalization, or vulnerability such as personal characteristics (e.g., language barriers); relationships barriers to accessing care (e.g., children in a household with migrants or homeless parents, parents’ occupation, or education); or environmental situations that provide limited control of opportunities for health (e.g., attending public school, neighborhood environment) [26].

### Comparators

Children in the non-exposed group as defined by the authors (will depend on the exposure group under evaluation).

### Outcomes

We will consider studies that assess healthcare delivery (e.g., access to appropriate care, and adherence to best practices) for children with injury. We will evaluate healthcare provided in the acute setting (i.e., pre-hospital, emergency department, and in-patient care). Studies on post-acute rehabilitation services will be considered in a separate review. Studies reporting on the influence of SDH inequities for clinical outcomes (e.g., mortality, disabilities, morbidity) or resource utilization (e.g., length of stay in hospital, costs) only (without assessing healthcare delivery) will not be considered.

### Study designs

We will include observational (i.e., retrospective and prospective cohorts, case–control studies) and experimental (i.e., randomized controlled trials, quasi-experimental studies) designs. We will exclude reviews, editorial articles, or reports if they do not present original data on the exposure-outcome associations of interest. Systematic reviews will be used to identify eligible studies not found by our search strategy. There will be no language or date restrictions. Articles in languages other than English or French will be translated using online translation tools for study selection and translators for data extraction.

## Data sources

We will systematically search the three following databases: PubMed, Excerpta Medica database (EMBASE), Cumulative Index to Nursing and Allied Health Literature (CINAHL), PsycINFO, Web of Science, and Academic Search Premier from their inception to a maximum of 6 months prior to submission for publication. We will also manually screen references of identified studies to find potentially relevant articles not retrieved using our search strategy. We will search the grey literature using Google Scholar.

## Search strategy

We will develop our search strategies in collaboration with a scientific librarian using an iterative process according to the Peer Review of Electronic Search Strategies (PRESS) guidelines [27] (Additional file 2). PubMed will be searched first to revise and improve the preliminary search strategy. The approved search strategy will be applied to EMBASE, CINAHL, PsycINFO, Web of Science, and Academic Search Premier thereafter. We will search for articles comprehensively, avoiding specific SDH-related keywords to ensure inclusivity and avoid limitations. The search strategy will then be limited to the combinations of keywords and controlled vocabulary on the themes of disparities (“disparity”, “health disparity”, “inequity”, and “health inequity”); trauma (“injuries”, “fractures”, and “trauma”); and pediatrics (“pediatric”, “child”, “infant”, “adolescent”, “youth”, and “young”). We will limit our search to articles that clearly identify SDH-related differences in access to and quality of care as disparities or inequities.

## Reference management

The articles from the various databases will be imported and merged into EndNote 20 software (Version X9.3.3, Thomson Reuters, New York City, 2018). All duplicates between databases will be either automatically or manually removed and the most recent version retained. The list of unique articles will be exported to Covidence systematic review software (Veritas Health Innovation, Melbourne, Australia) [28] for study screening.

## Selection process

Two content experts will first independently screen 5% of the identified unique articles to pilot selection based on the eligibility criteria described above. The pilot phase will be repeated until an acceptable agreement is reached (kappa > 0.7) [29]. The two reviewers will then independently screen all the unique articles based on titles and abstracts. The studies that both reviewers agree should not be included will be disregarded by default. The studies selected for inclusion by at least one of the reviewers or that did not provide sufficient information to allow evaluation merely based on the title or the abstract will be considered potentially eligible. The two reviewers will then independently evaluate the full texts of potentially eligible studies to determine eligibility for final inclusion. We will contact authors of studies with insufficient or unclear information for final decision-making at this stage. We will disregard the studies whose authors we were unable to contact after three attempts. For studies excluded at this stage, reasons for exclusion will be documented. In the event of any disagreement, the two reviewers will attempt to reach a consensus, and if necessary, a third reviewer will be called upon to arbitrate.

## Data collection process and data items

Data will be extracted by two independent reviewers using a standard data extraction form along with a detailed instruction manual developed and pilot-tested by our research team. We will retrieve information on study characteristics (first author, year of publication, country of study population, data sources and period covered, settings, data sources, and study design); the characteristics of the population (total sample size, age range, and injury types and mechanisms); the PROGRESS-Plus factors studied; the characteristics of exposed and comparison groups (type and frequency); the characteristics of the outcomes studied (type, frequency in the exposed and comparison groups; type of effect measure (e.g., odds ratio, relative risk, mean difference); crude and adjusted effect measures and their 95% confidence intervals); and adjustment variables. We will conduct a pilot extraction phase using three studies and repeat iteratively on further studies until an acceptable agreement is reached. In case of disagreements, we will attempt to reach a consensus among reviewers or consult a third reviewer when necessary. The authors of studies with missing or unclear information will be contacted as described above. In case of failure, data will be considered missing.

## Risk of bias in individual studies

Two reviewers will independently assess the risk of bias of included studies using the appropriate risk of bias assessment tool according to the study designs. We will use the Risk Of Bias In Non-randomized Studies-of Exposure (ROBINS-E) tool to assess the risk of bias in observational studies [30]. The ROBINS-E tool comprises seven domains of bias: confounding, selection of participants in the study, classification of exposures, departures from intended exposures, missing data, measurement of outcomes, and selection of the reported result [30]. Each of these bias domains and overall risk-of-bias will be rated as low, moderate, high risk-of-bias, or no information. If we identify any experimental studies, we will use the revised Cochrane Risk-of-Bias Tool (RoB 2) [31]. This tool covers five bias domains including bias arising from the randomization process, bias due to deviations from intended interventions, bias due to missing outcome data, bias in measurement of the outcome, and bias in selection of the reported result [31]. Both tools will be piloted on a random sample of 5% of the included studies to ensure consistency among reviewers. Any disagreements will be resolved by discussion between the two reviewers or by arbitration with a third party when necessary.

## Data synthesis

We will describe the study selection using a PRISMA flowchart. The extracted data will be synthesized in narrative form first, describing the studies and the PECOS elements (i.e., populations, exposures, comparators, outcomes, and study designs). For each outcome of interest, we will synthesize risk of bias assessments graphically according to each domain of bias and overall risk of bias, separately for experimental and observational studies.

If sufficient and appropriate data is available in at least three studies retained, we will conduct meta-analyses for each outcome of interest using R version 4.2.1 [32]. Pooled effect estimates and 95% confidence intervals will be calculated using random effects models. Publication bias will be explored using funnel plots [33]. We will assess heterogeneity using the I^2^ index [33].

## Subgroup and sensitivity analyses

To explore unexplained heterogeneity, if the number of studies is sufficient, we will conduct subgroup analyses for the following factors, identified by our project advisory committee: age (0–5, 6–14, 15–19; categories defined on consultation with advisory committee members); last year of data collection; geographical region (North America, South America, Europe, Asia, Africa, and Australia); type and mechanisms of injuries; World Bank income categories (lower-middle, upper-middle); and risk of bias (low, medium, and high).

Mechanisms of injury will be based on the International Classification of Diseases, tenth revision (ICD-10) criteria used by the American College of Surgeons, i.e., motor vehicle collisions, falls, struck by/against, other transport, and firearm/cut-pierce) [24, 25].

Types of injuries will be based on the American College of Surgeons Trauma Quality Improvement Program cohorts [34] and our previous work [35]: blunt multisystem injuries (Abbreviated Injury Scale (AIS) ≥ 3 in at least two body regions); traumatic brain injuries (intracranial lesions and Glasgow Coma Scale (GCS) 13–15 (mild), GCS 9–12 (moderate), or GCS 3–8 (severe)); spinal cord injuries (AIS codes 640200.3–640276.6, 640400.3–640468.5, 640600.3–640668.5, 630600.3–630638.4 except 630612.2 and 630614.3); solid organ injuries (blunt or penetrating injuries of the liver, spleen, kidney, or pancreas); and orthopedic fractures (fractures of the upper or lower extremities, pelvic ring, or spine not including spinal cord).

However, if information in the included articles is lacking or deviates from the above definition, we will form subgroups according to the authors’ definitions.

## Discussion

The findings of this review will advance knowledge on SDH-related inequities in pediatric injury care that clinicians and policymakers can use to design better care systems that offer equitable access to high-quality care to all children and adolescents after injury. However, this review has some limitations. Despite our intention to conduct a comprehensive review by including all the PROGRESS-Plus framework factors, we anticipate that we will not be able to conduct meta-analyses for some factors because of insufficient studies. Similarly, we expect heterogeneity in inclusion criteria, definitions of exposure and outcomes across studies, and insufficient studies to fully assess heterogeneity in results. We will disseminate our findings through infographic summaries distributed to clinical organizations, presentations to clinicians, healthcare administrators, and researchers (e.g., conferences, seminars, clinical rounds), and publication in a peer-reviewed journal.

### Supplementary Information

**Additional file 1.** PRISMA-P 2015 Checklist.

**Additional file 2: Table 1.** Search strategy in PubMed. **Table 2.** Search strategy in EMBASE. **Table 3.** Search strategy in CINAHL. **Table 4.** Search strategy in PsycINFO. **Table 5.** Search strategy in Web of Science. **Table 6.** Search strategy in Academic Search Premier.

**Additional file 3: Table 1.** Inclusion and exclusion criteria.

## Data Availability

Not applicable.

## Abbreviations

AIS: Abbreviated Injury Scale
CINAHL: Cumulative Index to Nursing and Allied Health Literature
EMBASE: Excerpta Medica Database
GCS: Glasgow Coma Scale
ICD-10: International Classification of Diseases, tenth revision
PECOS: Population, exposure, comparison, outcomes, and study design
PRESS: Peer Review of Electronic Search Strategies
PRISMA-P: Preferred Reporting Items for Systematic Reviews and Meta-Analyses-Protocols
PROGRESS: Place of residence, race/ethnicity, occupation, gender, religion, education, socioeconomic status, and social capital framework
PROSPERO: International Prospective Register of Systematic Reviews
ROB: Risk of bias
ROBINS-E: Risk of Bias in Non-randomized Studies-of Exposure
SDH: Social determinants of health
SES: Socioeconomic status
