# Supplementary material for: Social determinants of health and disparities in pediatric trauma care: protocol for a systematic review and meta-analysis
Source: Syst Rev. 2024 Mar 22;13:94. doi: 10.1186/s13643-024-02510-7 (PMC10958897; doi:10.1186/s13643-024-02510-7)
Supplement: Supplementary file 2 — Additional file 2: Table 1. Search strategy in PubMed. Table 2. Search strategy in EMBASE. Table 3. Search strategy in CINAHL. Table 4. Search strategy in PsycINFO. Table 5. Search strategy in Web of Science. Table 6. Search strategy in Academic Search Premier. [file 13643_2024_2510_MOESM2_ESM.docx]

**Additional file 2.** Preliminary search strategy

**Table 1:** Search strategy in PubMed

| **Research** | **Concepts** | **Search Strategy** |
| --- | --- | --- |
| #1 | Healthcare disparity  (Controlled vocabulary) | "Health Equity"[Mesh] OR "Health Inequities"[Mesh] OR "Healthcare Disparities"[Mesh] |
| #2 | Healthcare disparity  (Free text) | Disparit*[Tiab] OR Inequit*[Tiab] OR Equit*[Tiab] OR Inequalit*[Tiab] |
| #3 | Total Health disparity | #1 OR #2 |
| #4 | Pediatric  (Controlled vocabulary) | "Child"[Mesh] OR "Adolescent"[Mesh] OR "Pediatrics"[Mesh] OR "Infant"[Mesh] |
| #5 | Pediatric  (Free text) | Adolescen*[Tiab] OR Baby[Tiab] OR Babies[Tiab] OR Child*[Tiab] OR Boy[Tiab] OR Boys[Tiab] OR Girl*[Tiab] OR Paediatric*[Tiab] OR Kid[Tiab] OR Kids[Tiab] OR Newborn*[Tiab] OR Infant*[Tiab] OR Pediatric*[Tiab] OR Toddler*[Tiab] OR Young*[Tiab] OR Youth*[Tiab] OR Juvenile*[Tiab] OR Teen*[Tiab] |
| #6 | Total pediatric | #4 OR #5 |
| #7 | Trauma  (Controlled vocabulary) | "injuries"[MeSH Subheading] OR "wounds and injuries"[MeSH] |
| #8 | Trauma  (Free vocabulary) | fracture*[Tiab] OR trauma*[Tiab] OR injur*[Tiab] OR wound*[Tiab] |
| #9 | Total Trauma | #7 OR #8 |
| #10 | Overall | #3 AND #6 AND #9 |

**Table 2:** Search strategy in EMBASE

| **Research** | **Concepts** | **Search strategy** |
| --- | --- | --- |
| #1 | Health disparity | 'health disparity'/exp OR 'health care disparity'/exp OR 'health equity'/exp OR (‘equit*' OR ‘disparit*’ OR ‘inequit*’ OR inequalit*):ab,ti,kw |
| #2 | Trauma | 'injury'/exp OR (‘injur*’ OR 'fracture*' OR ‘trauma*’ OR ‘wound*’):ab,ti,kw |
| #3 | Pediatric | 'child'/exp OR 'adolescent'/exp OR ‘pediatric emergency medicine’/exp OR ('p$ediatric*' OR 'child*' OR 'youth*' OR 'young*' OR 'juvenile*' OR 'newborn*' OR 'adolescen*' OR 'boy*' OR 'girl*' OR 'baby' OR ‘babies’ OR 'kid*' OR ‘toddler*' OR 'teen*'):ab,ti,kw |
| #4 | Overall | #1 AND #2 AND #3 |

**Table 3:** Search strategy in CINAHL (EBSCO)

| Research | Concepts | Search Strategy |
| --- | --- | --- |
| #1 | Healthcare disparity  (Controlled vocabulary) | (MH "Health Inequities") or (MH “Healthcare Disparities") |
| #2 | Healthcare disparity  (Free text) | TI (disparit* or inequit* or inequalit* or equit*) or AB ( disparit* or inequit* or inequalit* or equit*) |
| #3 | Total Healthcare disparity | #1 OR #2 |
| #4 | Pediatric  (Controlled vocabulary) | (MH “Infant+”) or (MH " Child+") or (MH " Adolescent+") |
| #5 | Pediatric  (Free text) | TI (Adolescen* OR Baby OR Babies OR Child* OR Boy OR Boys OR Girl* OR Paediatric* OR Kid OR Kids OR Newborn* OR Infant* OR Pediatric* OR Toddler* OR Young* OR Youth* OR Juvenile* OR Teen*) or AB (Adolescen* OR Baby OR Babies OR Child* OR Boy OR Boys OR Girl* OR Paediatric* OR Kid OR Kids OR Newborn* OR Infant* OR Pediatric* OR Toddler* OR Young* OR Youth* OR Juvenile* OR Teen*) |
| #6 | Total pediatric | #4 OR #5 |
| #7 | Trauma  (Controlled vocabulary) | (MH “Trauma+") or (MH “wounds and injuries+") |
| #8 | Trauma  (Free vocabulary) | TI (trauma* or injur*) or AB (trauma* or injur*) |
| #9 | Total Trauma | #7 OR #8 |
| #10 | Overall | #3 AND #6 AND #9 |

**Table 4:** Search strategy in PsycINFO (OVID)

| **Research** | **Concepts** | **Search strategy** |
| --- | --- | --- |
| #1 | Health disparity | exp health disparities/ or (equit* or disparit* or inequit* or inequalit*).ti,ab,id |
| #2 | Trauma | exp injuries/ or (injur* or fracture* or trauma* or wound*).ti,ab,id |
| #3 | Pediatric | exp pediatrics/ or (p$ediatric* or child* or youth* or young* or juvenile* or newborn* or adolescen* or boy* or girl* or baby or babies or kid* or toddler* or teen*).ti,ab,id |
| #4 | Overall | #1 AND #2 AND #3 |

**Table 5:** Search strategy in Web of Science

| **Research** | **Concepts** | **Search strategy** |
| --- | --- | --- |
| #1 | Health disparity | TS=(disparit* OR equit* OR inequit* OR inequalit*) |
| #2 | Trauma | TS=(injur* OR fracture* OR trauma* OR wound*) |
| #3 | Pediatric | TS=(child* OR youth* OR young* OR juvenile* OR newborn* OR adolescen* OR boy* OR girl* OR baby OR babies OR kid* OR pediatric* OR paediatric* OR toddler OR teen*) |
| #4 | Overall | #1 AND #2 AND #3 |

**Table 6:** Search strategy in Academic Search Premier (EBSCO)

| **Research** | **Concepts** | **Search Strategy** |
| --- | --- | --- |
| #1 | Health disparity  (Controlled vocabulary) | DE "HEALTH equity" |
| #2 | Health disparity  (Free text) | TI (disparit* or inequit* or inequalit* or equit*) OR AB (disparit* or inequit* or inequalit* or equit*) OR KW (disparit* or inequit* or inequalit* or equit*) |
| #3 | Total Health disparity | #1 OR #2 |
| #4 | Pediatric  (Controlled vocabulary) | (DE “Children”) OR (DE “Teenagers”) OR (DE "Pediatrics") |
| #5 | Pediatric  (Free text) | TI (Adolescen* OR Baby OR Babies OR Child* OR Boy OR Boys OR Girl* OR Paediatric* OR Kid OR Kids OR Newborn* OR Infant* OR Pediatric* OR Toddler* OR Young* OR Youth* OR Juvenile* OR Teen*) or AB (Adolescen* OR Baby OR Babies OR Child* OR Boy OR Boys OR Girl* OR Paediatric* OR Kid OR Kids OR Newborn* OR Infant* OR Pediatric* OR Toddler* OR Young* OR Youth* OR Juvenile* OR Teen*) or KW (Adolescen* OR Baby OR Babies OR Child* OR Boy OR Boys OR Girl* OR Paediatric* OR Kid OR Kids OR Newborn* OR Infant* OR Pediatric* OR Toddler* OR Young* OR Youth* OR Juvenile* OR Teen*) |
| #6 | Total pediatric | #4 OR #5 |
| #7 | Trauma  (Controlled vocabulary) | (DE “Wounds and injuries") |
| #8 | Trauma  (Free vocabulary) | TI (trauma or injur*) or AB (trauma or injur*) or KW (trauma or injur*) |
| #9 | Total Trauma | #7 OR #8 |
| #10 | Overall | #3 AND #6 AND #9 |
