# Supplementary material for: Social determinants of health and disparities in pediatric trauma care: protocol for a systematic review and meta-analysis
Source: Syst Rev. 2024 Mar 22;13:94. doi: 10.1186/s13643-024-02510-7 (PMC10958897; doi:10.1186/s13643-024-02510-7)
Supplement: Supplementary file 3 — Additional file 3: Table 1. Inclusion and exclusion criteria. [file 13643_2024_2510_MOESM3_ESM.docx]

**Additional file 3.** Inclusion and exclusion criteria

Table 1: Inclusion and exclusion criteria

| **PECOS**  **elements** | **Questions related to eligibility criteria** | **Answers** |
| --- | --- | --- |
| **Population** | 1. Question 1?  - Is the study related to trauma?  - Is the study related to children or adolescents <19 years? | **YES,** if the study is related to children or adolescents <19 years with trauma;  **NO**, if  -adults / age >19 years old;  -not physical trauma-related;  -burns  **UNCLEAR**, if you cannot code YES or NO |
| **Exposure(s) /**  **Comparator (s)** | 2. Question 2?  - Does the study make the comparison based on at least one of the PROGRESS-Plus framework factors? | **YES,** if the studied factor(s) is/are PROGRESS factor(s) (place of residence, race/ethnicity, occupation, gender, religion, education, socioeconomic status, and social capital) or Plus factor(s) (age, disability, etc.);  **NO**, if no factor or factors other than specified above;  **UNCLEAR**, if you cannot code YES or  NO |
| **Outcome(s)** | 3. Question 3?  Did the authors report at least a care delivery outcome (access to care, adherence to best practices)? | **YES,** if outcomes are related to care delivery;  **NO**, if only clinical outcomes reported (mortality, disabilities, morbidity);  **UNCLEAR**, if you cannot code YES or NO |
| **Study design(s)** | 4. Question 4?  Is the study observational or experimental? | **YES,** if the study is observational or experimental;  **NO**, if reviews, editorial articles, or reports;  **UNCLEAR**, if you cannot code YES or NO |
